# Clinical efficacy of baloxavir marboxil versus oseltamivir in kidney transplant recipients with influenza

**DOI:** 10.1128/spectrum.02954-24

**Published:** 2025-05-22

**Authors:** Jiali Jiang, Jianping Wang, Wenjing Hou, Bangqin Hu, Pan Chen, Fang Zeng, Yan Zhang, Qing Qian, Kuifen Ma

**Affiliations:** 1 Department of Clinical Pharmacy, The First Affiliated Hospital, Zhejiang University School of Medicine 26441 , Hangzhou, Zhejiang, China; 2 Department of Pharmacy, The First Affiliated Hospital of Zhejiang Chinese Medical University (Zhejiang Provincial Hospital of Chinese Medicine) 74723, Hangzhou, China; 3 Department of Pharmacy, The Second People’s Hospital of Shanxi Province, Taiyuan, China; 4 National Alliance of Transplant Pharmacists, Zhejiang, China; 5 Department of Pharmacy, Beijing Friendship Hospital Affiliated to Capital Medical University, Beijing, China; 6 Department of Pharmacy, The Second Affiliated Hospital of Chongqing Medical University 585250, Chongqing, China; 7 Department of Pharmacy, The First Affiliated Hospital, Sun Yat-sen Univeristy, Guangzhou, China; 8 Department of Pharmacy, Union Hospital, Tongji Medical College, Huazhong University of Science, Wuhan, China; 9 Department of Pharmacy, The First Affiliated Hospital of Soochow University, Suzhou, China; 10 Department of Pharmacy, The First People’s Hospital of Changzhou, Changzhou, China; 11 Zhejiang Provincial Key Laboratory for Drug Evaluation and Clinical Research, Hangzhou, China; Oklahoma State University College of Veterinary Medicine, Stillwater, Oklahoma, USA

**Keywords:** baloxavir marboxil, oseltamivir, kidney transplant recipients, influenza symptom, fever

## Abstract

Kidney transplant recipients (KTRs) are at high risk for severe influenza and its complications. The effectiveness of baloxavir marboxil (hereafter baloxavir) in infected KTRs is still unknown. In this multicenter retrospective study conducted at eight Grade IIIA hospitals in China, KTRs diagnosed with influenza between October 2023 and March 2024 were enrolled. Of the 246 patients assessed, 117 met the inclusion criteria. There were 59 patients who received baloxavir and 58 patients who received oseltamivir. Patients receiving baloxavir had a longer time from symptom onset to treatment (≤48 h, 59.3% versus 79.3%; *P* = 0.019). Nevertheless, in comparison with those who were administered oseltamivir, they exhibited no statistically significant difference in time to alleviation of influenza symptoms [4 (2–6) versus 5 (3–7); *P* = 0.054], duration of fever [3 (2–4) versus 3 (2–4); *P* = 0.347], or viral clearance [6 (40.0%) versus 10 (58.8%); *P* = 0.479]. However, among patients who received antivirals within 48 h, baloxavir showed a significant correlation with fever resolution when compared with oseltamivir [2 (2, 3) versus 3 (2–4); *P* = 0.030]. Meanwhile, among patients who received antivirals after 48 h, a remarkable improvement of symptom alleviation in 5 days was observed in the baloxavir-treated group as compared to the oseltamivir-treated group [18 (75.0%) versus 2 (16.7%); *P* = 0.001]. In conclusion, compared to oseltamivir, patients may derive benefits from baloxavir treatment within 48 h, but especially after 48 h from symptom onset.

**IMPORTANCE:**

The multicenter cohort study is the first to compare the clinical efﬁcacy of baloxavir with oseltamivir in influenza kidney transplant recipients (KTRs). The results showed that there were fewer influenza symptoms but a longer time for viral shedding in KTRs compared to non-immunosuppressed patients. No significant difference regarding time to alleviation of influenza symptoms and fever resolution between baloxavir and oseltamivir was found, which is consistent with CAPSTONE-1, whereas influenza KTRs who received baloxavir could have a shorter fever duration and symptom alleviation in 5 days compared to the oseltamivir-treated group. Our findings may provide guidance for influenza therapy in KTRs, solid organ transplant recipients, and immunocompromised patients.

## INTRODUCTION

The coronavirus disease 2019 (COVID-19) pandemic has changed the transmission dynamics of seasonal influenza and other respiratory infectious diseases (1, 2). Owing to the reduced pathogenicity of SARS-CoV-2 and enhanced herd immunity that may be attributed to natural infection and COVID-19 vaccination (3, 4), all restrictive measures have been scrapped in China since 8 January 2023. Previously, the Chinese Center for Disease Control and Prevention (China CDC) has forecasted that compared with the 2017–2019 seasons, the 2021–2022 influenza outbreak would be longer and blunter once the public health and social measures (PHSM) are fully lifted (5). In reality, during the 2023–2024 season, the number of influenza-like illness (ILI) and laboratory-confirmed influenza (LCI) cases in China increased significantly, reaching the highest level in nearly 10 years (6).

Influenza, an acute respiratory infectious disease caused by the influenza type A and B viruses, is a common respiratory infection in solid organ transplant (SOT) recipients that may lead to severe complications, including pneumonia, high hospitalization, and impaired allograft outcomes (7, 8). In Finland, between 1995 and 2017, there was a fivefold increase in LCI cases and a more than fourfold increase in the risk of hospitalization due to influenza among kidney transplant recipients (KTRs) compared to the general population (9). Although neuraminidase inhibitors (NAIs) such as oseltamivir, peramivir, and zanamivir are generally effective against human influenza infection, the emergence of resistance to these drugs highlights the need for new antiviral medications (10, 11). Baloxavir marboxil (hereafter baloxavir), the prodrug of baloxavir acid, is a polymerase inhibitor targeting the endonuclease activity of the virus PA protein. It was first licensed in Japan and the USA in 2018, and in China in 2021 for the treatment of influenza A or B (12, 13). Clinical evidence has confirmed that baloxavir was superior to oseltamivir in reducing viral load either in patients with acute influenza infection (14, 15) or outpatients with uncomplicated influenza (CAPSTONE-2) (16). Whereas immunosuppressed patients were excluded in these studies, the effectiveness of baloxavir and oseltamivir in these populations requires further investigation. So far, the effects of baloxavir on patients with creatinine clearance (CrCl) below 50 mL/min or severe renal impairment have not been evaluated (17), and there were no clinical data available in KTRs.

The aim of this study was to explore the pathogenetic characteristics of immunosuppressed KTRs with influenza infection and to compare the efficacy of baloxavir and oseltamivir in the treatment of influenza in a cohort of KTRs.

## MATERIALS AND METHODS

### Study design and participants

This retrospective study was conducted between October 2023 and March 2024 at eight Grade IIIA hospitals in China. In this cohort study, KTRs were enrolled according to the following inclusion criteria: (i) received either baloxavir or oseltamivir; (ii) confirmed influenza infection with positive RT-PCR or rapid antigen test detected on nasopharyngeal swab; (iii) no other respiratory viruses or mycoplasma infection; (iv) no bacterial or fungal infections.

### Treatments and definition

Typically, patients in the baloxavir group were treated with a single oral dose of baloxavir: 40 mg for those weighing <80 kg (except two patients taking 20 mg) and 80 mg for those weighing over 80 kg. Patients in the oseltamivir group received oral oseltamivir twice daily at a dosage of 30 or 75 mg (drug dosing based on GFR). Patients with no clinical improvement may receive additional doses as assessed by nephrologists and clinical pharmacists. Symptom alleviation was defined as the composite symptom score being graded as 0 or 1. Fever resolution was identified as multiple measurements taken several hours apart showed a body temperature of <37.3°C.

### Study methods and statistical analysis

The primary objective of this study was to compare the time to alleviation of symptoms between baloxavir- and oseltamivir-treated groups. Secondary objectives were to determine time from antivirals to fever resolution and rate of viral RNA and antigen clearance. Subset analyses were performed as follows: patients with confirmed influenza A, patients with confirmed influenza B, patients who received antiviral treatment within 48 h of symptom onset, and patients who received antiviral treatment after 48 h of symptom onset.

Continuous variables were compared between the baloxavir- and oseltamivir-treated groups by *t*-test or Wilcoxon test, while categorical variables were compared using χ² test or Fisher’s exact test. The Kaplan–Meier curves of time to alleviation of symptoms and fever duration were constructed, and the generalized Wilcoxon test was used to compare the differences between the groups. A multivariate logistic regression analysis was performed to evaluate potential confounding factors associated with a shorter time to symptom alleviation. Factors were incorporated into the multivariate models if their *P*-values were below 0.1 or they were deemed to have clinical potential to confound the outcomes of time to symptom alleviation. Statistical analysis was performed using SPSS v.25 and GraphPad Prism v.8.2.1, and *P* < 0.05 was considered statistically significant.

## RESULTS

### Baseline characteristics

**Fig 1:**
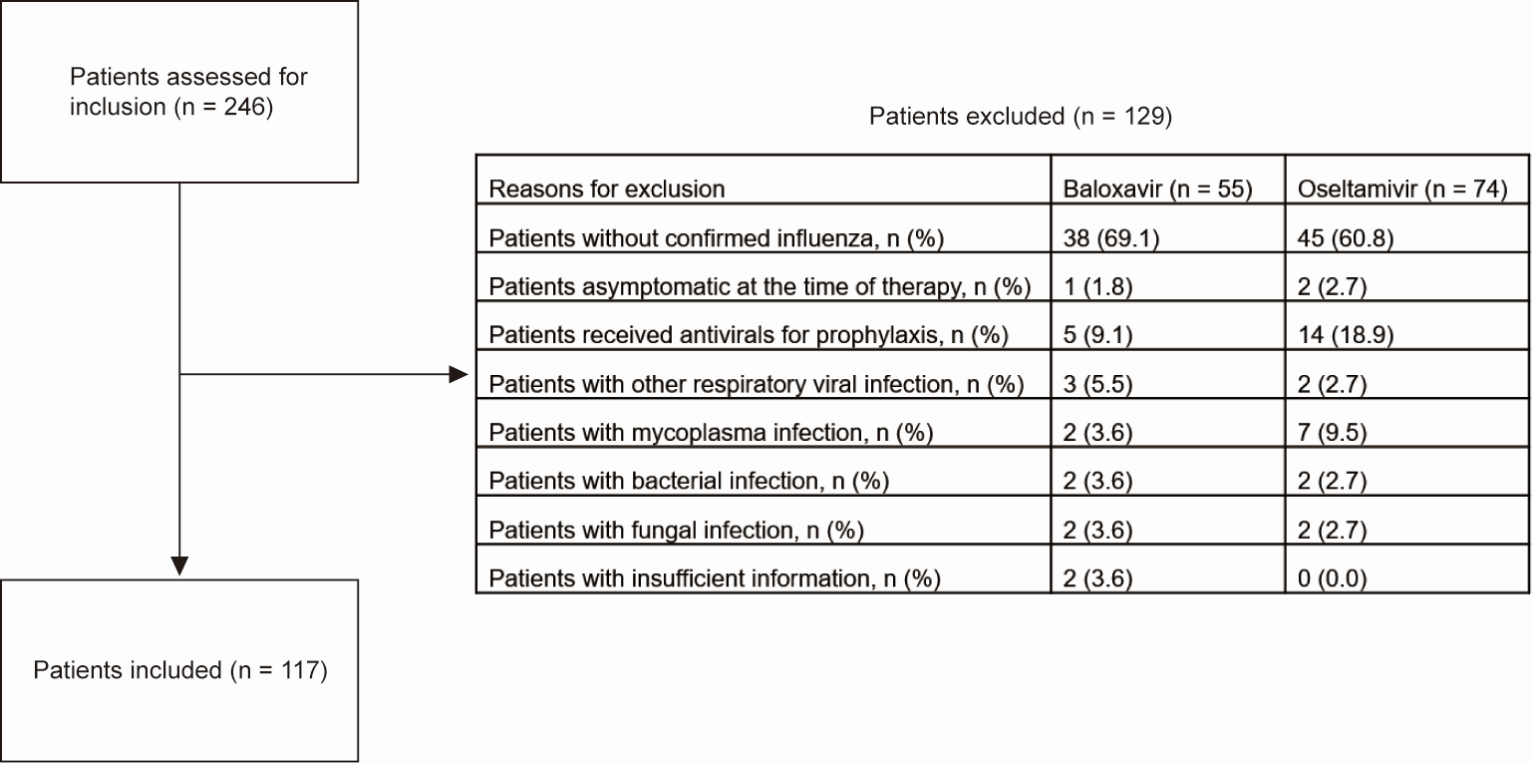
Reasons for exclusion.

**Fig 2:**
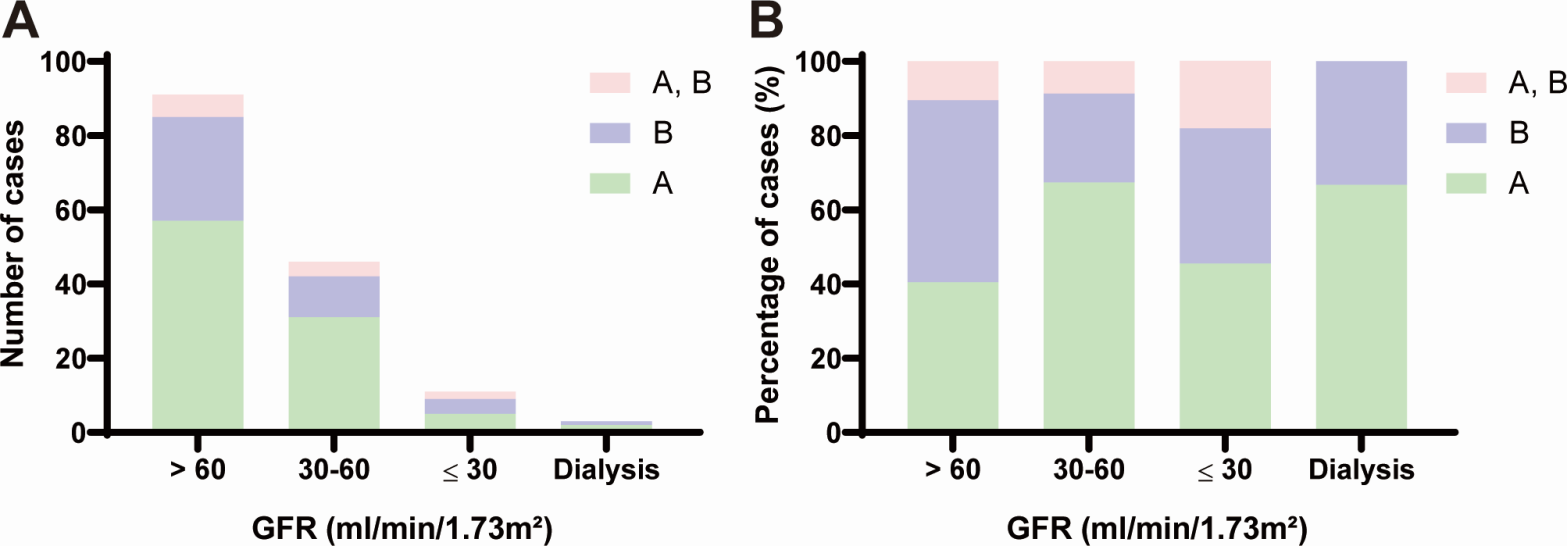
Prevalence of influenza type based on GFR. (**A**) The number of cases. (**B**) The percentage of cases. Green bar: influenza type A, purple bar: influenza type B, pink bar: mixed infection.

**Fig 3:**
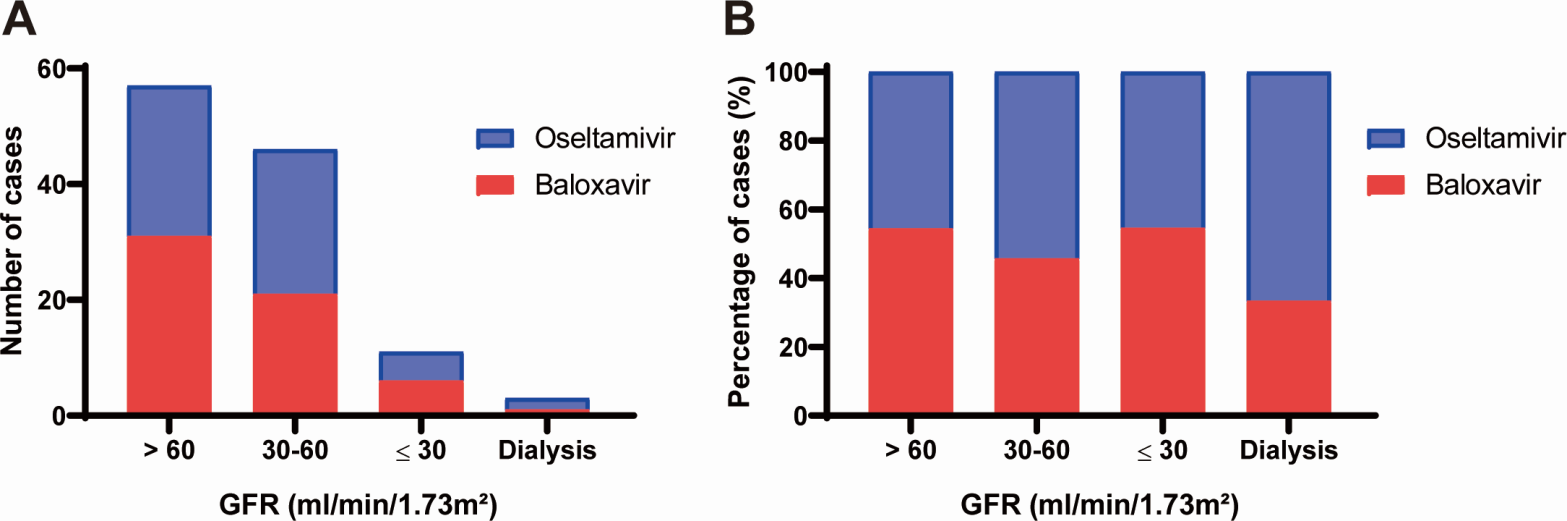
Comparison of antivirals based on GFR. (**A**) The number of cases. (**B**) The percentage of cases.

Of the 246 patients assessed, 117 patients met the inclusion criteria (Fig. 1). Figures 2 and 3 depicted the influenza types and antiviral treatment based on GFR. Except for patients with GFR 30–60 mL/min/1.73 m^2^, types A and B were almost evenly distributed in patients with varying degrees of renal dysfunction. Thirty-one (67.4%) of the 46 patients with GFR 30–60 mL/min/1.73 m^2^ were infected with type A, while 11 (23.9%) were infected with type B. Patients with different GFR showed no preference in choosing baloxavir or oseltamivir.

There were 59 patients who received baloxavir and 58 patients who received oseltamivir. Baseline demographics of the cohort are detailed in Table 1. Briefly, patients who received baloxavir were more likely to take mycophenolate mofetil [58 (98.3%) versus 39 (67.2%); *P* < 0.001] rather than mizoribine [1 (1.7%) versus 18 (31.0%); *P* < 0.001], and less likely to have diabetes [7 (11.9%) versus 21 (36.2); *P* = 0.002], hypertension [42 (71.2%) versus 50 (86.2%); *P* = 0.048], and obesity [22.0 ± 3.9 versus 23.4 ± 3.9; *P* = 0.045]. Differences between patients who received baloxavir and oseltamivir in influenza type (mix infection in 1.7% of patients who received baloxavir versus 19.0% of patients who received oseltamivir) and time from symptom onset to treatment (≤48 h in 59.3% of patients who received baloxavir versus 79.3% of patients who received oseltamivir) were observed (Table 2).

**TABLE 1:** Baseline demographics

Characteristic	Baloxavir (*n* = 59)	Oseltamivir (*n* = 58)	*P*-value
Age, years, median (range)	42 (12–69)	42 (18–66)	0.371^*a*^
Male, *n* (%)	40 (67.8)	35 (60.3)	0.401^*b*^
Weight, kg, mean	61.2 ± 15.3	66.0 ± 13.0	0.067^*a*^
<80 kg, *n* (%)	50 (84.8)	49 (84.5)	0.969^*b*^
BMI,^*c*^ mean	22.0 ± 3.9	23.4 ± 3.9	0.045^*a*^
Comorbidities, *n* (%)			
Hypertension	42 (71.2)	50 (86.2)	0.048^*b*^
Diabetes	7 (11.9)	21 (36.2)	0.002^*b*^
Current smoker	8 (13.6)	4 (6.9)	0.377^*b*^
Chronic lung disease	4 (6.8)	3 (5.2)	1.000^*b*^
Malignancy	1 (1.7)	7 (12.1)	0.063^*b*^
Immunosuppression at admission, *n* (%)			
Tacrolimus	52 (88.1)	53 (91.4)	0.563^*b*^
Cyclosporine	6 (10.2)	5 (8.6)	0.774^*b*^
Mycophenolate mofetil	57 (96.6)	39 (67.2)	<0.001^*b*^
mTOR inhibitor	4 (6.8)	8 (13.8)	0.211^*b*^
Mizoribine	1 (1.7)	18 (31.0)	<0.001^*b*^
Steroids	54 (91.5)	54 (93.1)	0.749^*b*^

^*a*^ Unpaired *t*-test.

^*b*^ χ² test.

^*c*^ BMI, body mass index.

**TABLE 2:** Clinical characteristics of influenza infection in kidney transplant recipients

Characteristic	Baloxavir (*n* = 59)	Oseltamivir (*n* = 58)	*P*-value
Influenza virus type, *n* (%)			
A	36 (61.0)	25 (43.1)	0.004^*c*^
B	22 (37.3)	22 (37.9)	
Mixed infection	1 (1.7)^*a*^	11 (19.0)^*a*^	
Body temperature, °C, mean	38.2 ± 0.9	38.1 ± 1.0	0.374^*d*^
Composite symptom score^*b*^, median (IQR)	6 (4–8)	8 (4–11)	0.102^*d*^
Prior influenza vaccination, *n* (%)	2 (3.4)	2 (3.5)	0.986^*e*^
Time from symptom onset to treatment, hours, *n* (%)			
≤48 h	35 (59.3)	46 (79.3)	0.019^*d*^
>48 h	24 (40.7)	12 (20.7)	

^*a*^ The difference between the groups was statistically significant.

^*b*^ Composite symptom score (range: 0–21) was used to evaluate the severity of seven influenza symptoms (feverishness or chills, cough, sore throat, nasal congestion, headache, muscle or joint pain, and fatigue) on a 4-point scale: 0, no symptoms; 1, mild symptoms; 2, moderate symptoms; and 3, severe symptoms. Higher score indicating more severe illness (14, 16, 18).

^*c*^ Fisher’s exact test.

^*d*^ Wilcoxon test.

^*e*^ χ² test.

### Clinical outcome

**Fig 4:**
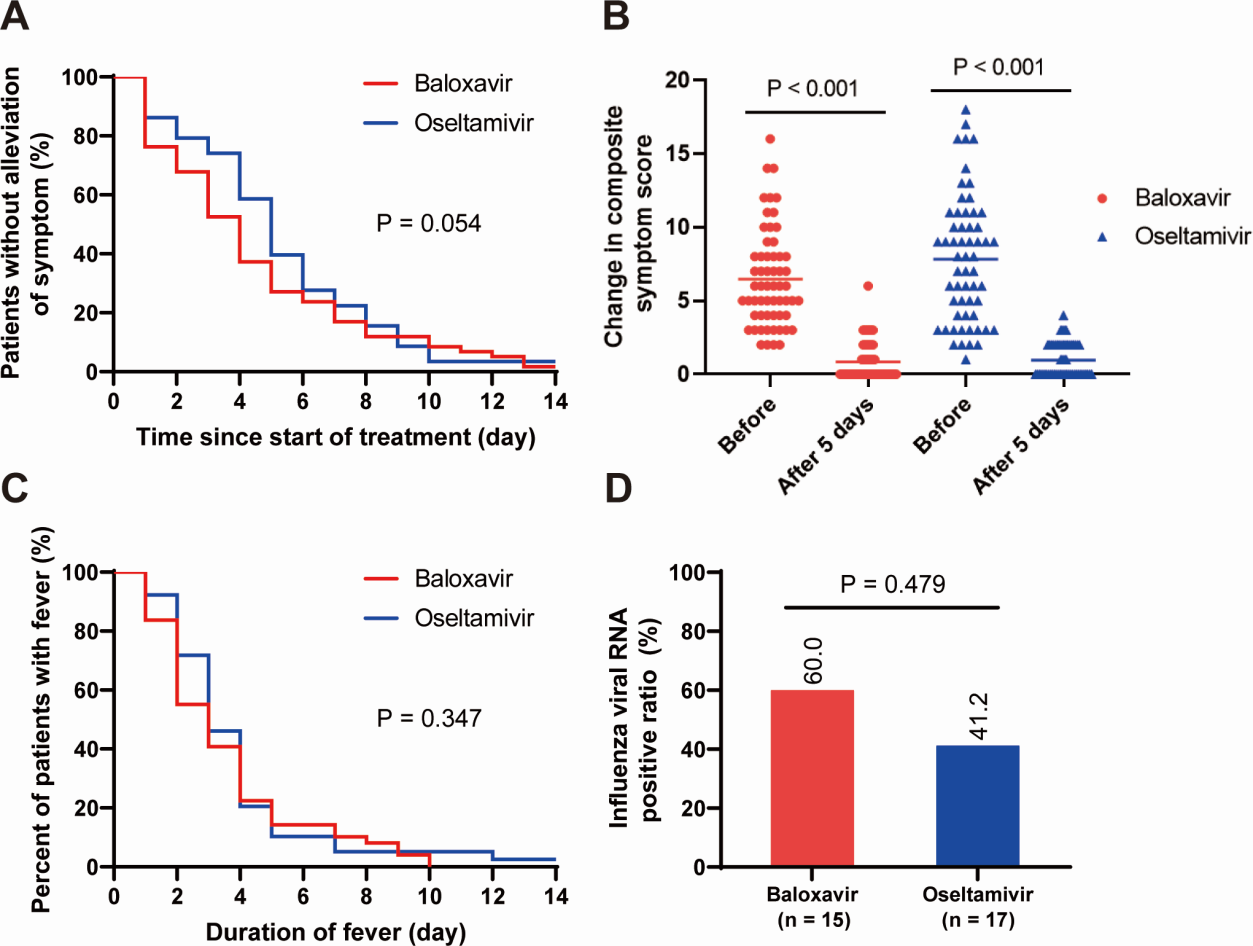
Comparison of clinical outcomes between baloxavir and oseltamivir treatment groups. (**A**) The Kaplan–Meier curves of time to alleviation of symptoms. The median time to alleviation of symptoms was 4 days in the baloxavir group and 5 days in the oseltamivir group. (**B**) Changes in composite symptom scores before and 5 days after antiviral treatment. (**C**) The Kaplan–Meier curves of the duration of fever. The median duration of fever was both 3 days in the baloxavir and oseltamivir groups. (**D**) Positive ratio of viral RNA or antigen at 5 days after antiviral therapy.

The Kaplan–Meier curves for time to alleviation of symptoms and fever duration in baloxavir and oseltamivir groups were shown in Fig. 4A and C. The median time to alleviation of symptoms was 4 days in the baloxavir group and 5 days in the oseltamivir group. The median duration of fever in patients who received baloxavir and oseltamivir was both 3 days. No difference in time to alleviation of symptoms (*P* = 0.054) or duration of fever (*P* = 0.347) was found between patients who received baloxavir and those who received oseltamivir, according to the generalized Wilcoxon test. Also, patients who received baloxavir had no significant difference in symptom alleviation in 5 days [44 (74.6%) versus 35 (60.3%); *P* = 0.100] or fever resolution in 5 days [42 (85.7%) versus 35 (89.7%); *P* = 0.570] compared with those who received oseltamivir (Table 3). Additionally, the paired *t*-test was used to compare the changes in composite symptom scores before and 5 days after antiviral treatment (Fig. 4B). In contrast, a significant difference in composite symptom scores was observed in both baloxavir- and oseltamivir-treated groups (*P* < 0.001). Among the patients retested for influenza viral RNA or antigen after antiviral treatment, 9 (60.0%) were positive in the baloxavir group and 7 (41.2%) in the oseltamivir group (Fig. 4D). In the multivariate logistic regression analysis, no independent clinical factor associated with time to symptom alleviation was identified (Table 4).

**TABLE 3:** Comparison of clinical outcomes between baloxavir- and oseltamivir-treated groups

	Baloxavir	Oseltamivir	*P*-value
Symptom alleviation in 5 days, *n* (%)	*n* = 59 44 (74.6)	*n* = 58 35 (60.3)	0.100^*a*^
Time from antivirals to symptom alleviation, days, median (IQR)	*n* = 59 4 (2–6)	*n* = 58 5 (3–7)	0.054^*b*^
Fever resolution in 5 days, *n* (%)	*n* = 49 42 (85.7)	*n* = 39 35 (89.7)	0.570^*a*^
Time from antivirals to fever resolution, days, median (IQR)	*n* = 49 3 (2–4)	*n* = 39 3 (2–4)	0.347^*b*^
Influenza viral RNA or antigen clearance, *n* (%)	*n* = 15 6 (40.0)	*n* = 17 10 (58.8)	0.479^*c*^

^*a*^ χ² test.

^*b*^ Wilcoxon test.

^*c*^ Fisher’s exact test.

**TABLE 4:** Multivariate logistic regression analysis of factors associated with time to symptom alleviation

Factor	Odds ratio (OR)	95% CI	*P*-value
BMI	0.931	0.833–1.040	0.205
Hypertension	0.999	0.342–2.917	0.998
Diabetes	2.382	0.871–6.513	0.091
Malignancy	1.075	0.213–5.414	0.930
Mycophenolate mofetil	1.833	0.426–7.890	0.416
Mizoribine	0.623	0.137–2.839	0.541
Mixed infection	1.167	0.301–4.527	0.824
Therapy with baloxavir	2.298	0.902–5.855	0.081
≤48 h from symptom onset to treatment	1.550	0.655–3.668	0.319

In a subgroup analysis of patients who received antivirals within 48 h, baloxavir was significantly correlated with fever resolution compared with oseltamivir [2 (2, 3) versus 3 (2–4); *P* = 0.030]. Meanwhile, among patients who received antivirals after 48 h, a significant improvement of symptom alleviation in 5 days was observed in the baloxavir-treated group compared with the oseltamivir-treated group [18 (75.0%) versus 2 (16.7%); *P* = 0.001] (Table 5). No significant difference in time to symptom alleviation or fever resolution was noted in the other subgroups.

**TABLE 5:** Subgroup analyses based on influenza types and time from symptom onset to treatment

Subgroup of patients with confirmed influenza A			
	Baloxavir (*n* = 36)	Oseltamivir (*n* = 25)	*P*-value
Symptom alleviation in 5 days, *n* (%)	*n* = 36 29 (80.6)	*n* = 25 18 (72.0)	0.540^*a*^
Time from antivirals to symptom alleviation, days, median (IQR)	*n* = 36 3 (1–5)	*n* = 25 5 (1.5–6)	0.261^*b*^
Fever resolution in 5 days, *n* (%)	*n* = 31 26 (83.9)	*n* = 18 18 (100.0)	0.143^*a*^
Time from antivirals to fever resolution, days, median (IQR)	*n* = 31 3 (2–4)	*n* = 18 3 (2–4)	0.672^*b*^
Influenza viral RNA or antigen clearance, *n* (%)	*n* = 10 6 (60.0)	*n* = 6 2 (33.3)	0.608^*a*^

Subgroup of patients with confirmed influenza B			
	Baloxavir (*n* = 22)	Oseltamivir (*n* = 22)	*P*-value
Symptom alleviation in 5 days, *n* (%)	*n* = 22 14 (63.6)	*n* = 22 13 (59.1)	1.000^*a*^
Time from antivirals to symptom alleviation, days, median (IQR)	*n* = 22 4.5 (2–8)	*n* = 22 5 (3.75–8.25)	0.570^*b*^
Fever resolution in 5 days, *n* (%)	*n* = 17 15 (88.2)	*n* = 13 10 (76.9)	0.628^*a*^
Time from antivirals to fever resolution, days, median (IQR)	*n* = 17 3 (2–4.5)	*n* = 13 4 (2–6)	0.470^*b*^
Influenza viral RNA or antigen clearance, *n* (%)	*n* = 5 0 (0.00)	*n* = 6 4 (66.7)	0.061^*a*^

Subgroup of patients who received antivirals within 48 h of symptom onset			
	Baloxavir (*n* = 35)	Oseltamivir (*n* = 46)	*P*-value
Symptom alleviation in 5 days, *n* (%)	*n* = 35 26 (74.3)	*n* = 46 35 (76.1)	1.000^*a*^
Time from antivirals to symptom alleviation, days, median (IQR)	*n* = 35 3 (1–7)	*n* = 46 5 (3–7)	0.078^*b*^
Fever resolution in 5 days, *n* (%)	*n* = 32 30 (93.8)	*n* = 33 31 (93.9)	1.000^*a*^
Time from antivirals to fever resolution, days, median (IQR)	*n* = 32 2 (2, 3)	*n* = 33 3 (2–4)	0.030^*b*^
Influenza viral RNA or antigen clearance, *n* (%)	*n* = 9 4 (44.4)	*n* = 14 8 (57.1)	0.680^*a*^

Subgroup of patients who received antivirals after 48 h of symptom onset			
	Baloxavir (*n* = 24)	Oseltamivir (*n* = 12)	*P*-value
Symptom alleviation in 5 days, *n* (%)	*n* = 24 18 (75.0)	*n* = 12 2 (16.7)	0.001^*a*^
Time from antivirals to symptom alleviation, days, median (IQR)	*n* = 24 4 (2.25–6)	*n* = 12 6 (4–7.75)	0.203^*b*^
Fever resolution in 5 days, *n* (%)	*n* = 17 12 (70.6)	*n* = 6 3 (50.0)	0.621^*a*^
Time from antivirals to fever resolution, days, median (IQR)	*n* = 17 4 (4–7)	*n* = 6 5 (4.75–8.25)	0.251^*b*^
Influenza viral RNA or antigen clearance, n (%)	*n* = 6 2 (33.3)	*n* = 3 2 (66.7)	0.524^*a*^

^*a*^ Fisher’s exact test.

^*b*^ Wilcoxon test.

## DISCUSSION

The present study is the first to compare the clinical efficacy of baloxavir with oseltamivir in KTRs with influenza. Previously, Memoli et al. (19) demonstrated that immunosuppressed patients (including patients with malignancy, hematopoietic stem cell transplants, or immunosuppressive therapy) with influenza exhibited fewer clinical symptoms but longer viral shedding. Similarly, we found that influenza symptoms of KTRs at baseline were lower than those in the CAPSTONE-1 and CAPSTONE-2 studies (14, 16). Also, a longer time for fever resolution in KTRs was observed, regardless of the equal results (3 days) in baloxavir and oseltamivir groups in the full cohort analysis. In addition, Memoli et al. (19) observed a mean viral shedding time of 19.0 days in immunosuppressed patients versus 6.4 days in non-immunosuppressed patients. In the current study, 6 (40.0%) of the baloxavir group and 10 (58.8%) of the oseltamivir group obtained viral RNA or antigen clearance in 5 days, suggesting a longer time for viral shedding in KTRs.

The active sites of the influenza’s PA subunits in influenza A and B are nearly identical, indicating an equal effectiveness of baloxavir against influenza A and B (20, 21). Resistance testing was not available in this study since giving routine reporting was not a standard of care in most hospitals. The China CDC estimated that resistance to oseltamivir between 2 October 2023 and 31 March 2024 was 0.03% (22), indicating little effect in patients who received oseltamivir in our study. Subgroup analyses with influenza A H3N2 or B virus infections in the CAPSTONE-2 study showed that more rapid reductions in viral load were seen with baloxavir treatment compared to oseltamivir in patients with uncomplicated influenza (16). In contrast, no significant difference was found in our study regarding viral clearance of influenza KTRs between baloxavir and oseltamivir. This result may be associated with the fact that a majority of patients did not retest for viral RNA because their influenza symptoms were largely relieved and no serious complications were found.

Unfortunately, studies on the efficacy of baloxavir and oseltamivir in the treatment of influenza in SOT recipients or immunosuppressed patients are scarce. Although a subgroup analysis was not performed, a retrospective study including immunosuppressed patients demonstrated that baloxavir resulted in faster hypoxia resolution than oseltamivir in hospitalized patients with influenza A (23). In our study, no significant difference regarding time to alleviation of influenza symptoms and fever resolution between baloxavir and oseltamivir was found, which is consistent with CAPSTONE-1 (14). However, the subgroup analysis conducted on influenza KTRs who received antivirals within 48 h of symptom onset revealed that patients who received baloxavir had a shorter fever duration than the oseltamivir-treated group (*P* = 0.030). This finding differs from that of a retrospective study showing no significant difference in time for fever resolution between baloxavir and oseltamivir in patients who received antivirals within 48 h of symptom onset (23). Among patients who received antivirals after 48 h of symptom onset, 18 patients (75.0%) in the baloxavir group and 2 patients (16.7%) in the oseltamivir group achieved symptomatic alleviation within 5 days (*P* = 0.001).

There are several limitations in this study. As a retrospective study, the available data were collected from the electronic medical record and during follow-up visits. The real-world study across multiple centers hardly allowed standardization of the frequency of influenza detection to assess for viral clearance. Among patients receiving oseltamivir therapy, 34 (58.6%) derived from one hospital in Northern China, which might lead to potential selection bias. In addition, since the current study was conducted in both hospitalized patients and outpatients, the time from antiviral treatment to fever resolution, which is a critical endpoint in this study, was analyzed in days rather than hours after onset. Finally, a small subset of baloxavir patients (8.5%) could have received up to two doses of oseltamivir while waiting for the results of influenza detection. Consequently, the findings of these analyses should be interpreted with caution.

### Conclusion

This was a multicenter cohort study on KTRs with influenza comparing the effectiveness of baloxavir and oseltamivir treatment. In patients who received antivirals within 48 h of symptom onset, baloxavir treatment contributed to a shorter fever duration than oseltamivir treatment. There was no significant difference between the efficacy of baloxavir and oseltamivir in KTRs with influenza types A and B. Further large prospective multicenter clinical studies are needed to validate this conclusion.
